# Seizure prophylaxis in the neuroscience intensive care unit

**DOI:** 10.1186/s40560-018-0288-6

**Published:** 2018-03-05

**Authors:** Sushma Yerram, Nakul Katyal, Keerthivaas Premkumar, Premkumar Nattanmai, Christopher R. Newey

**Affiliations:** 1 Department of Neurology, University of Missouri, 5 Hospital Drive, CE 540, Columbia, MO 65211 USA; 2 Department of Biological Sciences, University of Missouri, Columbia, MO 65211 USA; 3 Cleveland Clinic, Cerebrovascular Center, 9500 Euclid Avenue, Cleveland, OH 44195 USA

**Keywords:** Seizure prophylaxis, Critically ill patients, Anti-epileptic drugs, Continuous electroencephalography

## Abstract

**Background:**

Seizures are a considerable complication in critically ill patients. Their incidence is significantly high in neurosciences intensive care unit patients. Seizure prophylaxis with anti-epileptic drugs is a common practice in neurosciences intensive care unit. However, its utility in patients without clinical seizure, with an underlying neurological injury, is somewhat controversial.

**Body:**

In this article, we have reviewed the evidence for seizure prophylaxis in commonly encountered neurological conditions in neurosciences intensive care unit and discussed the possible prognostic role of continuous electroencephalography monitoring in detecting early seizures in critically ill patients.

**Conclusion:**

Based on the current evidence and guidelines, we have proposed a presumptive protocol for seizure prophylaxis in neurosciences intensive care unit. Patients with severe traumatic brain injury and possible subarachnoid hemorrhage seem to benefit with a short course of anti-epileptic drug. In patients with other neurological illnesses, the use of continuous electroencephalography would make sense rather than indiscriminately administering anti-epileptic drug.

## Background

Seizures are a considerable complication in critically ill patients. Their incidence is higher in neurosciences intensive care unit (NSICU) patients [1]. Seizure prophylaxis with anti-epileptic drugs (AED) is a common practice in NSICU. However, its utility in patients without clinical seizure, with an underlying neurological injury is somewhat controversial. AEDs for seizure prophylaxis are used in various acute neurological insults including traumatic brain injury (TBI), epidural hemorrhage (EDH), subdural hemorrhage (SDH), aneurysmal subarachnoid hemorrhage (SAH), intracerebral hemorrhage (ICH), brain neoplasms, ischemic stroke, cavernoma and arteriovenous malformation (AVM), cerebral venous sinus thrombosis (CVST), posterior reversible encephalopathy syndrome (PRES), and meningitis. The underlying risk for seizure varies significantly in all these disease states. In this article, we have reviewed the evidence for seizure prophylaxis in commonly encountered neurological conditions in NSICU and discussed the possible prognostic role of continuous electroencephalography (CEEG) monitoring in detecting early seizures in critically ill patients.

## Traumatic brain injury

Post traumatic seizures (PTS) in TBI patients are classified as early PTS, with seizure occurring within 7 days of injury and late PTS, with seizure occurring after 7 days of injury [1, 2]. According to a civilian study [1], the incidence rate for early PTS ranges between 4 and 25%, whereas the incidence rate for late PTS ranges between 9 and 42% [1]. Studies have identified multiple risk factors increasing the likelihood of PTS in TBI patients [3]. Penetrating injuries are associated with highest incidence of PTS with more than 50% of patients developing seizures over 15 years [1] (Table 1).

**Table 1:** Risk factors for seizure in patients with traumatic brain injury

Risk factors for early PTS [3]	Risk factors for late PTS [3]
• Glasgow coma scale < 10 • Penetrating brain injuries • Acute intracerebral hematoma • Acute subdural hematoma • Younger age • Loss of consciousness • Post traumatic amnesia lasting > 30 min • Chronic alcoholism	• Early PTS • Acute intracerebral hematoma • Brain contusion • Loss of consciousness • Post traumatic amnesia lasting > 24 h • Age > 65 at the time of injury

The risk factors for early and late PTS include [3]:

### Seizure prophylaxis in traumatic brain injury

Given the high incidence of seizures in TBI patients, AEDs are frequently used as prophylactic therapy. Temkin et al. [4] conducted a randomized, double-blinded, placebo-controlled trial to evaluate the effects of phenytoin (20 mg/kg load) on early and late PTS. A significant reduction was observed in the incidence of early PTS in phenytoin group from 14.2 to 3.6% (*p* < 0.001) whereas, no significant reduction was noted in the incidence of late PTS with phenytoin use. Unfortunately, patients with seizures were included in the prophylaxis group making the data difficult to interpret. No significant side effects or mortality differences were observed between phenytoin and placebo groups [5]. Significant impairment was noted on neuropsychological testing in phenytoin group patients at 1 month time after starting therapy, which later was not apparent after 1 year of injury [6].

In another RCT, the effects of valproate on early and late PTS were studied. The trial later compared valproate (20 mg/kg load) to phenytoin (20 mg/kg load) for prevention of early PTS valproate to placebo for prevention of late PTS [7]. It was reported that the rate of early PTS was low and similar in both valproate (4.5%) and phenytoin (1.5%) groups [7]. The rate of late PTS was also similar, being high in both groups (valproate 16%, phenytoin 15%). However, a significantly higher mortality rate was observed in valproate group (13.4%) as compared to phenytoin (7.2%) [7]. No potential added benefits and higher mortality rates suggest that valproate should not be used for seizure prophylaxis. A meta-analysis study conducted in 2001 showed that out of all AED, only phenytoin and carbamazepine were effective in reducing early PTS. No AED was found to be effective in reducing late PTS [8].

Levetiracetam has been studied for seizure prophylaxis [9]. Levetiracetam has no known drug interactions, excellent bioavailability and relatively safer pharmacological profile; these make it somewhat ideal for prophylactic use [9]. In a study to compare effectiveness of levetiracetam (500 mg BD) to that of phenytoin (historical controls) for preventing seizures in patients with TBI, no differences were observed in seizure activity between the two (levetiracetam 6.7 and phenytoin 0%, *p* = 0.556) [10]. Strikingly, an increased incidence of EEG abnormalities (seizure tendency with epileptiform activity) was noted in levetiracetam group (*p* = 0.003). No difference in EEG findings of seizures (*p* = 0.556) was seen in the two groups [10]. In another study comparing seizure prophylaxis with levetiracetam (1500 mg BD) versus phenytoin (20 mg/kg load followed by 5 mg/kg/d maintenance), no significant differences were seen in early seizure rates (phenytoin 3 of 18 vs levetiracetam 5 of 34; *p* = 1.0) [11]. However, levetiracetam group reportedly had lower disability rating scale scores at 3 and 6 months (*p* = 0.006 and *p* = 0.037, respectively) and higher extended Glasgow outcome scale scores at 6 months [11] (Table 2).

**Table 2:** Brain trauma guidelines for management of TBI patients

According to latest brain trauma foundation guidelines (2016) [75]
• Phenytoin is recommended for prevention of early PTS and it should be used for first 7 days after TBI [75].
• Phenytoin or valproate use is not recommended for prevention against late PTS [75].
• Given its safety profile levetiracetam could be a potential alternative to phenytoin for prophylaxis against early PTS. Presently there is not enough corroborative evidence to support its use over phenytoin [75].

### Current recommendations

#### Aneurysmal subarachnoid hemorrhage

Abnormal seizure like moments is common in patients with aneurysmal subarachnoid hemorrhage (aSAH) [12, 13]. Their incidence can be as high as 26% [12, 13]. They may be associated more with the posturing event during rupture of aneurysm than actual seizures [12, 13]. Clinical seizures occur in about 1 to 7% of patients with aSAH and typically are manifestations of rerupture of an unsecured aneurysm [14, 15]. The incidence rate for nonconvulsive seizures in patients with aSAH is reportedly 8 to 18% [16–18]. Risk factors increasing the likelihood of seizures in aSAH patients are [12, 13] (Table 3).

**Table 3:** Risk factors for seizures in aSAH patients

Risk factors for seizures in aSAH patients [12, 13]
• Prior seizures
• History of HTN
• Intraparenchymal hemorrhage
• Infarction
• Middle cerebral artery aneurysm
• Thickness of aSAH clot
• Rebleeding
• Poor neurological grade
• Intervention: endovascular coiling associated with a lower risk of seizure compared to open craniotomy for clipping

### Seizure prophylaxis in aneurysmal subarachnoid hemorrhage

AED prophylaxis for aSAH is somewhat controversial [19]. Limited randomized controlled trials (RCT) justifying the prophylactic use and serious adverse effects of AED make the decision even more challenging [19, 20]. Seizures in acutely ill patients with aSAH can lead to additional injury or rebleeding from an unsecured aneurysm, which make AED prophylaxis critical in some cases.

In a study to assess the utility of phenytoin (900–1100 mg load followed by 300 mg/d) for seizure prophylaxis in patients with aSAH, investigators observed a low seizure incidence of about 5.4% after 2.4 years of follow-up [21]. In a retrospective study, undertaken to compare different duration of phenytoin (1 g load followed by 300 mg/d) prophylaxis (3 days prophylaxis versus 14 days prophylaxis), no significant differences were observed in seizure incidences during hospitalization (1.9 vs 1.3%, *p* = 0.6) and after a follow-up period of 3 to 12 months (4.6 vs 5.7%, *p* = 0.6) [22]. A significant reduction was noticed in incidence of adverse drug reactions with 3 days prophylaxis (0.5 vs 8.8%, *p* = 0.002), which indicates that a 3-day regimen is a superior treatment protocol [22]. This study was further scrutinized to assess the relationship between phenytoin exposure and harm by quantifying phenytoin burden and estimating its impact on outcomes [20]. Phenytoin burden was identified as an independent predictor for poor functional outcome at 14 days (OR per quartile 1.5%, 95% CI 1.2–1.9) and for poor cognitive outcome at 3 months (*p* = 0.003) [20]. A study estimating the impact of AED on outcomes by analyzing data from four RCTs reported that the use of AED in patients with aSAH was associated with poor a 3-month outcomes with worse Glasgow outcome scale (OR 1.56, *p* = 0.003), vasospasm (OR 1.87, *p* < 0.001), neurological deterioration (OR 1.61, *p* < 0.001), cerebral infarction (OR 1.33, *p* = 0.04), and fever (OR 1.36, *p* = 0.03) [19].

A possible risk for drug-drug interaction exist in patients taking phenytoin and nimodipine, a calcium channel blocker commonly used to counter vasospasm in patients with aSAH [23]. A single-dose pharmacokinetic study of nimodipine in patients on chronic enzyme inducing AED showed a mean decrease of 70% (*p* < 0.01) in plasma nimodipine concentration in patients taking enzyme inducing AED and an increase of 50% in plasma nimodipine concentration in patients taking valproate [23].

Given its relatively safer profile, there is a growing interest in support of using levetiracetam for seizure prophylaxis [9]. A retrospective study comparing phenytoin (15–20 mg/kg load; 13.7 days) to levetiracetam (500 mg BD; 3.6 days) in aSAH patients reported a higher seizure incidence in levetiracetam group (8.3 vs 3.4%) [24]. A lower incidence of poor outcome (death or nursing home discharge) was noticed in levetiracetam group (16 vs 24%, *p* = 0.06) [24]. Nonetheless, the concerns like lack of loading dose of levetiracetam, a shorter course of therapy renders the study statistically insignificant (Table 4).

**Table 4:** Neurocritical Care Society guidelines and 2012 American Heart Association/American stroke Association guidelines for management of patients with aSAH

As per the 2011 Neurocritical Care Society guidelines [71] and 2012 American Heart Association/American stroke Association guidelines [76]
• Phenytoin is not recommended routinely for seizure prophylaxis after SAH [71].
• Other AED may be considered for seizure prophylaxis [71].
• A short course is preferable (3–7 days) in case prophylaxis is needed [71].
• CEEG monitoring should be used in patients who failed to improve or have poor grade SAH [71].
• Prophylactic use of AED can be considered in immediate post hemorrhagic period [76].
• Long-term use of AED can be considered for patients with known risk factors for delayed seizure disorder, such as prior seizure, intracerebral hematoma, intractable hypertension, infarction, or aneurysm at the middle cerebral artery [76].

### Current recommendations

#### Brain neoplasm

Seizures are commonly reported in patients with brain neoplasms [25, 26]. The incidence rate of seizures at or before the time diagnosis of brain tumor diagnosis ranges from 14 to 51%, whereas after the diagnosis, it varies between 10 and 45% [25].

Risk factors for seizures in patients with brain neoplasm vary according to [26]:

- Type of tumor: primary tumor > metastatic
- Grade of tumor: low-grade glioma > high-grade glioma
- Specific tumor types: dysembryoplastic neuroepithelial tumors > meningiomas

### Seizure prophylaxis in brain neoplasms

A meta-analysis study of five trials from 1999 through 2004 evaluated the efficacy of AED phenobarbital, phenytoin, and valproic acid versus placebo or no treatment for seizure prophylaxis in patients with brain neoplasm [27]. Four of the trials showed no significant benefits of seizure prophylaxis at 1 week (OR 0.91, CI 0.45–1.83) or at 6 months (OR 1.01, CI 0.51–1.98) in patients with brain neoplasm [27]. AED prophylaxis showed no preventive benefits for specific tumor pathologies, including primary glial tumors (OR 3.46, 95% CI 0.32–37.47), cerebral metastasis (OR 2.50, 95% CI 0.25–24.72), and meningiomas (OR 0.62, 95% CI 0.10–3.85) [27]. The cochrane review in 2008 came to similar conclusions but did find higher incidence of adverse effects in patients on AEDs (NNH 3; RR 6.1, CI 1.1–34.63; *p* = 0.046) [28]. The American Academy of Neurology (AAN) introduced practice parameters for seizure prophylaxis in patients with brain neoplasms [25]. Based on results from 12 studies evaluating efficacy of AED phenobarbital, phenytoin and valproic acid over a median follow-up time of 5.4 to 19 months, it was concluded that AED prophylaxis had no significant preventive effect on either seizure incidence (OR 1.09, CI 0.63–1.89; *p* = 0.08) or seizure free survival (OR 1.03, CI 0.74–1.44; *p* = 0.9) [25]. Significant side effects were reported in the trials including rash (14%), nausea/vomiting (5%), encephalopathy (5%), myelosuppression (3%), ataxia, increased liver enzymes, and gum pain (5%) [25].

Guidelines for prophylactic use of AED in patients with metastatic brain neoplasms were derived from analysis of a RCT, evaluating the efficacy of AED phenobarbital, phenytoin versus no treatment [29, 30]. No significant differences were observed in seizure incidence between two groups in the trial [29]. It was concluded that seizure prophylaxis was not beneficial in patients with metastatic brain tumors [29]. Another RCT comparing the efficacy of short course AED prophylaxis with phenytoin for 7 days versus no prophylaxis in patients with intraparenchymal tumors showed no significant difference in seizure incidence between two groups (24 vs 18%; *p* = 0.51) [31]. However, significant adverse effects were reported in phenytoin group (18 vs 0%; *p* < 0.01) [31]. The most commonly reported side effects were thrombocytopenia, confusion, aphasia, decreased level of consciousness, nausea, vomiting, dry itchy skin, ataxia, and photophobia [31].

A meta-analysis review of 19 studies from 1979 through 2010 evaluated the efficacy of AED including phenytoin, valproic acid, carbamazepine, lamotrigine, and levetiracetam versus that of no treatment in patients undergoing resection of supratentorial meningioma [32]. No significant differences were observed in incidence of both early (1.4 vs 1.4%, *p* > 0.05) and late seizures (8.8 vs 9.0%, *p* > 0.05) in two groups. It was concluded that seizure prophylaxis with AED is not beneficial in patients undergoing supratentorial meningioma resection [32].

A retrospective study was conducted to evaluate the efficacy of levetiracetam (1000 to 3000 mg) for seizure prophylaxis in patients undergoing surgery for brain tumors [33]. A seizure incidence of 2.6% was reported in levetiracetam group 1 week following surgery, which was significantly lower than the previously known seizure incidence in patients who did not receive prophylactic AED (15–20%) [33]. 6.4% of patients receiving levetiracetam reportedly had progressive somnolence and reactive psychosis [33]. Another retrospective study was conducted to compare the efficacy of a 750-mg load dose of phenytoin to that of a 1000-mg load dose of levetiracetam, both followed by taper over 5 days for seizure prophylaxis in patients undergoing surgery for brain tumors [34]. No significant differences were observed in rate of postoperative seizures between the two groups (levetiracetam 2.5 vs phenytoin 4.5%, *p* = 0.66) [34].

### Current recommendations

**Table:** 

• AED are not recommended for routine prophylactic use in patients with newly diagnosed brain neoplasm, as they are not effective in preventing first seizure and have potential side effects [32].
• In patients with brain neoplasm, who never had seizure, discontinuation or taper of AED after first postoperative week is recommended. This is particularly beneficial in medically stable patient or in those experiencing side effects [32].
• Seizure prophylaxis is not considered beneficial in patients with metastatic brain tumors [29].
• Seizure prophylaxis with AED is not beneficial in patients undergoing supratentorial meningioma resection [32].

## Intracerebral hemorrhage

The risk of seizure is highest within first few days after ictus in patients with intracerebral hemorrhage. More than 50% of seizures occurs in the first 24 h [35–43]. The incidence of early seizures in patients with ICH is reported to be 28 to 31% on CEEG monitoring [38, 43]. Clinical seizures are seen from 5.5 to 24% of the patients with ICH [38, 43]. The underlying cause of early seizures is believed to be immediate metabolic and physical disturbances in brain following ICH [39, 40, 44]. Late seizures are seen less frequently in patients with ICH and are believed to be due underlying gliotic scarring [37, 39, 44]. Risk factors increasing the likelihood of seizures in patients with ICH are not well known because of limited clinical studies [37, 40, 41].

### Seizure prophylaxis in intracerebral hemorrhage

A prospective study conducted on 761 patients with nontraumatic, nonaneurysmal ICH, without seizure in first 24 h, grouped on the basis of ICH location showed significant decrease in incidence of early seizures in patients with lobar ICH receiving phenobarbital prophylaxis as compared to patients with no treatment (5.9 vs 13.6%) [41]. It was reported that early prophylaxis could be beneficial in patients with lobar ICH [41].

A placebo-controlled RCT evaluated the association between the use of AED and poor outcomes (severe disability or death) by using modified Rankin scale. AED was started in 23 patients (8%) without documented seizure. The use of AED was associated with poorer outcomes after adjustment for other known predictors of outcome after ICH (OR 6.83; CI 2.2–21.23; *p* = 0.001). It was concluded that prophylactic use of AED especially phenytoin was associated with poor outcomes in patients with acute ICH [45].

Another study reported poor outcomes with prophylactic AED therapy in patients with ICH. This study evaluated data from 98 patients with ICH taking phenytoin, levetiracetam, or both [46]. It was reported that phenytoin use was associated with more fever (*p* = 0.03), worse scores on National Institutes of Health Stroke Scale at 14 days (23 [9 to 42] versus 11 [4 to 23], *P* = 0.003), and worse scores on modified Rankin scale at 14 and 28 days and 3 months as compared to levetiracetam [46]. It was speculated that poor outcomes could be related to reportedly larger ICH volume in patients on phenytoin prophylaxis [46] (Table 5).

**Table 5:** AHA/ASA guidelines for management of patients with ICH

As per the AHA/ASA guidelines for management of ICH [72]
• Prophylactic use of AED is not recommended in patients with ICH [72].
• Clinical seizures should be treated with anti-epileptic drugs [72].
• Continuous EEG monitoring is probably indicated in ICH patients with depressed mental status out of proportion to the degree of brain injury [72].
• Patients with a change in mental status who are found to have electrographic seizures on EEG should be treated with anti-epileptic drugs [72].

### Current recommendations

#### Ischemic stroke

Ischemic stroke is the most common cause of seizure in elderly patients [47]. Incidence rate of seizure in ischemic stroke patients ranges from 4 to 23% [48]. Post stroke seizures can be classified into early seizures, occurring within 7 days of stroke and late seizure, occurring after 7 days of stroke [49, 50]. Early seizure occurs from 2 to 6% of stroke patients and is believed to be related to edema and cytotoxicity associated with ischemic insult [49, 51]. Late seizure occurs in 3–5% of stroke patients and is related to underlying gliosis and meningo cerebral scarring [49, 51].

A higher risk for seizure development exists in certain patients:

- Patients with hemorrhagic stroke or with hemorrhagic transformation are at higher risk of developing seizures (12.5%) as compared to ischemic stroke without hemorrhagic transformation (4.2%, *p* < 0.0001) [49, 51].
- Cortical involvement in stroke patients predisposes them to higher seizure risk (9.8%) as compared to those with subcortical involvement (3.8%, *p* < 0.005) [49, 51].
- Patients with involvement of more than one lobe are at higher risk (21.2%) as compared to those with single lobe involvement (5.2%) [49, 51].

### Seizure prophylaxis in ischemic stroke

Currently, there is limited corroborative data available to support the use of AED for seizure prophylaxis in post stroke patients.

### Current recommendations

**Table:** 

As per the AHA guidelines for management of patients with acute ischemic stroke (2013) [52] and malignant cerebral edema (2014) [53]:
• Prophylactic use of AED is not recommended in patients with ischemic stroke [52].
• Seizure prophylaxis in patients without seizures at presentation is not recommended [53].

## Postoperative craniotomy

Incidence of seizure in patients with craniotomy varies greatly and depends upon the type of procedure performed and underlying pathology [54]. Incidence rate of seizures in patients with post supratentorial craniotomy is estimated to be 15 to 20% [54]. Risk of seizure varies between 3 and 92% over a 5-year period post craniotomy [54].

### Seizure prophylaxis in postoperative patients

A retrospective study compared prophylactic use of phenytoin (*n* = 210; most common dosage 300 mg/d, range 200–800 mg/d) to that of levetiracetam (*n* = 105; most common dosage 1000 mg/d, range 500–3000 mg/d) in 315 patients, who underwent supratentorial neurosurgery for wide range of disease pathologies [55]. An early seizure incidence (within 7 days) of 1% was reported in levetiracetam group as compared to 4.3% (*p* = 0.17) in phenytoin group [55]. Incidence of late seizure (within 30 days) was 1.9% in levetiracetam group as compared to 5.2% in phenytoin group (*p* = 0.23) [55]. No significant difference was observed in incidence of postoperative seizures between the levetiracetam and phenytoin groups (0 vs 1.8%, *p* = 0.56) in patients with a history of preoperative seizures [55]. Patients on levetiracetam prophylaxis had significantly lower incidence of adverse effects as compared to those on phenytoin prophylaxis (1 vs 18%, *p* < 0.001) [55]. Both the AEDs were associated with low risk of early as well as late seizures. The safety profile of levetiracetam (fewer adverse effects) makes it somewhat ideal for prophylactic use in post neurosurgery patients.

### Current recommendations

**Table:** 

As per the cochrane review 2013 [54]:
• There is a limited evidence to support the prophylactic use of AED in post neurosurgery patients.

## Vascular lesions

### Cavernous and arteriovenous malformations

A recent prospective study evaluated a 5-year seizure risk in 368 patients with either cavernous malformation (CM) (*n* = 139) or arteriovenous malformation (AVM) (*n* = 229) [56]. Five-year seizure risk was reportedly higher in patients with AVM presenting with intracranial hemorrhage or focal neurologic deficit (ICH/FND) 23% (*n* = 119; 95% confidence interval (CI) 9–37%) compared to incidental AVM 8% (*n* = 40; 95% CI 0–20%) [56]. Risk of developing epilepsy in incidental AVM was reportedly 2% per person-year, annualized over 5 years [56].

Five-year risk of seizure in patients with CM presenting with focal neurological deficit or ICH was 6% (*n* = 38; 95% CI 0–14%) compared to incidentally found cavernoma 4% (*n* = 57; 95% CI 0–10%) [56]. Risk of developing epilepsy in incidental cavernoma was reportedly 0.9% per person-year, annualized over 5 years [56].

### Current recommendations

**Table:** 

As per the current ASA guidelines for management of patients with either cavernous or arteriovenous malformations [57]:
• Surgical or radiosurgical obliteration of AVM is generally considered effective in reducing seizure activity [57].
• Currently there are not enough studies available to formulate recommendations regarding type and duration of AED prophylaxis after treatment [57].

### Other conditions

#### Cerebral venous thrombosis (CVT)

Seizures occur in about 40% of patients with CVT [58].

Risk factors increasing likelihood of seizure in patients with CVT are [58]:

- Motor deficit (OR 5.8, CI 2.98–11.42, *p* < 0.001) [58]
- ICH (OR 2.8, CI 1.46–5.56, *p* = 0.002) [58]
- Cortical vein thrombosis (OR 2.9, CI 1.43–5.96, *p* = 0.003) [58]

Prophylactic use of AED in patients with CVT is somewhat controversial. As per the cochrane review (2014) [59], there is no evidence to support or refute the use of anti-epileptic drugs for the primary or secondary prevention of seizures related to intracranial venous thrombosis [59].

### Current recommendations

**Table:** 

As per the AHA guidelines for management of patients with acute CVT [58]:
• In absence of seizure, routine use of AED in patients with CVT is not recommended [58].

#### Posterior reversible leukoencephalopathy syndrome (PRES)

Seizures occur in up to 68.8% patients with PRES [60]. However, there is not enough literature evidence to support prophylactic use of AED in patients with PRES.

#### Meningitis

Seizures occur in up to 27% of patients with meningitis [61]. There is not enough literature evidence to support prophylactic use of AED in patients with meningitis.

Guidelines for prophylactic use of AED in various neurological conditions encountered in the neurocritical ICU are generally derived from corroborative evidence provided by various RCT. For the majority of conditions, currently, there are not enough studies available to formulate recommendations regarding type and duration of AED prophylaxis [57]. In conditions without definite recommendations, the decision regarding prophylactic use of AED is usually derived from physician’s personal belief and local practice trends. However, under such circumstances, an ideal approach should be diagnosing seizure or epileptiform abnormalities rather than prophylaxis.

## Recognizing seizures in neurocritical ICU

Seizures can be difficult to recognize in critically ill patients. Prompt recognition of nonconvulsive seizures (NCSz) or nonconvulsive status epilepticus (NCSE) is crucial. Certain clinical presentations can indicate the ongoing NCS [62], these are as follows:

- An apparently prolonged “postictal state” following generalized convulsive seizures or with prolonged reduction of alertness from an operative procedure or neurologic insult [62]
- Acute onset of impaired consciousness or fluctuating picture with episodes of normal mentation [62]
- Impaired mentation or consciousness with myoclonus of facial muscles or nystagmoid eye movements [62]
- Episodic blank staring, aphasia, automatisms (lip smacking, fumbling with fingers), perseverative activity [62]
- Aphasia without an acute structural lesion
- Other acutely altered behavior without other obvious etiology [62]

A high degree of clinical suspicion is required to recognize these clinical presentations [62]. Prompt recognition is critical as NCS are associated with high mortality rate of 33% [63]. However, clinical presentations are not always reliable in accurately predicting the ongoing NCS. In a retrospective review of 208 patients admitted to neurocritical ICU from emergency department (ED) with either acute transient neurological deficits, loss of consciousness (LOC), or unclear motor phenomena, 13.9% of the patients were incorrectly diagnosed with epileptic seizures, whereas in 15.6% of patients who were eventually admitted to neurocritical ICU, diagnosis of epilepsy was missed in the ED [64]. The most common factors associated with missing seizure diagnosis were no prior history of epilepsy, older age (mean 76.4 years), multimorbidities, CT showing cerebrovascular lesion, seizure description given by nonprofessionals, negative seizure phenomenon (e.g., aphasia, LOC, paresis), and lack of tongue biting [64].

A retrospective review study evaluated 52 video EEGs over 18-month period in a neurocritical ICU [65]. These EEGs were from patients with possible seizures due to motor phenomenon [65]. Fourteen patients (27%) were found to have epileptic seizures [65]. That included four focal motor status epilepticus, three focal clonic, three myoclonus, two generalized status epilepticus, one focal tonic, and one generalized tonic clonic [65]. Thirty-eight patients (73%) had nonepileptic events, out of which 12 patients (23%) had tremor like events, 7 (13.5%) had multifocal jerks, 7 (13.5%) had slow semi purposeful movements, and the rest 12 (23%) had “other movements” [65]. These studies indicate that diagnosing seizures based on clinical presentation alone can be misleading. CEEG monitoring can be helpful in such circumstances. In a study of 236 patients with coma and no overt seizure activity, 8% of the patients were found to have NCSE on CEEG monitoring [66].

## Role of CEEG monitoring in neuro ICU

Continuous electroencephalography (CEEG) monitoring is commonly used in critically ill patients. CEEG is tightly linked to cerebral metabolism and, thus, sensitive to changes in cerebral blood flow [67]. Several patterns on CEEG are of diagnostic and prognostic significance in patients with cerebral edema that lie on the ictal-interictal continuum [68]. EEG patterns that correlate with increased intracranial pressure (ICP) include focal slowing of underlying rhythms or global EEG suppression progressing to burst suppression or flat EEG [67]. CEEG monitoring provides real-time dynamic information about brain functioning and allows for detection of any early change in neurological status of a patient that may not be evident on neurological examination alone [67]. CEEG is also tightly linked to cerebral metabolism and thus sensitive to changes in cerebral blood flow [67]. CEEG monitoring can identify NCS and NCSE in critically ill patients. Evaluation for suspected NCS is the most common indication for CEEG [69]. Studies have reported that the use of CEEG monitoring in ICU patients at risk for NCS can change the treatment protocol in the majority of patients [70]. In a retrospective study, Kilbride et al. showed that CEEG monitoring leads to AED modifications in 52% of patients, which included therapy initiation in 14%, modification in 33%, and discontinuation in 5% [70]. The NCS 2011 guidelines advocated use of CEEG monitoring in patients who failed to improve or have poor grade SAH (low-quality evidence-strong recommendation) [71]. The AHA/ASA guidelines also supported use of CEEG monitoring in patients with depressed mental status out of proportion to the degree of brain injury (Class IIa; Level of Evidence:B) [72]. According to AHA/ASA guidelines for management of ICH, only patients with “Clinical seizures should be treated with anti-epileptic drugs (Class I; Level of Evidence:A)” [72]. AED may be initiated for these events, but as they carry a significant risk for serious adverse effects including rash (Stevens Johnson syndrome), hematological abnormalities, behavioral changes, drug-drug interactions [1], it is ideal to diagnose the seizure before initiating prophylactic therapy in such circumstances [1].

The duration of CEEG monitoring is also an important aspect in diagnosing seizures in neurocritical ICU. In a retrospective review, Claassen et al. [73] reviewed CEEG data of 100 patients and reported time to seizure duration [73]. Sixty percent of noncomatose patients had seizure during the first hour of monitoring, whereas 95% of noncomatose patients had seizure in first 24 h [73]. In comparison to noncomatose patients, 50% of comatose patients had seizure in the first hour and only 80% had seizure in the first 24 h [73]. Eighty-seven percent of comatose patients reportedly had seizure within 48 h of monitoring [73]. This study concluded that comatose patients may require monitoring longer than the usual 24 h for detection of their first electrographic seizure [73].

Regarding the management of patients on CEEG monitoring, generally, if NCS or NCSE are detected, most physicians recommend initiating treatment, although the management approach can be highly variable [74]. Physicians tend to treat NCSE more aggressively than NCS, with a trend toward lesser use of anti-convulsants like levetiracetam and more willingness to induce coma and intubation if necessary [74]. Given the limited literature evidence regarding treatment of NCS and NCSE for adult or pediatric patients, management is pretty much similar across age groups.

CEEG monitoring can be helpful in conditions lacking sufficient evidence to support the use of AED for prophylactic use. Until there is more data available to provide supporting evidence, a potential seizure prophylaxis protocol can be summarized as follows (Table 6):

**Table 6:** Seizure prophylaxis protocol in neuro-ICU

Seizure prophylaxis	Conditions
Definitive prophylaxis	• Severe TBI (7 days)
Probable prophylaxis	• Unsecured aneurysm in SAH • Elevated intracranial pressure (ICP) and concern for poor compliance
Possible/no prophylaxis	• ICH • AVM • Cavernoma • Brain neoplasm • Malignant ischemic stroke • Postoperative craniotomy • Meningitis • Cerebral venous sinus thrombosis (CVST) • PRES

## Conclusion

The data for prophylactic use of AEDS in neuro critically ill patients lacks robustness. Patients with severe TBI and possible SAH seem to benefit with a short course of AED. In patients with injury to their brain, the use of CEEG would make sense rather than indiscriminately administering AED. Only observed seizures should be treated in such patients.

## Abbreviations

AED: Anti-epileptic drugs
AVM: Arteriovenous malformation
CEEG: Continuous electroencephalography
CVST: Cerebral venous sinus thrombosis
ED: Emergency department
EDH: Epidural hemorrhage
ICH: Intracerebral hemorrhage
LOC: Loss of consciousness
NCSE: Nonconvulsive status epilepticus
NCSz: Nonconvulsive seizures
NSICU: Neurosciences intensive care unit
PRES: Posterior reversible encephalopathy syndrome
PTS: Post traumatic seizures
SAH: Aneurysmal subarachnoid hemorrhage
SDH: Subdural hemorrhage
TBI: Traumatic brain injury
